# Fluorescence excitation-scanning hyperspectral imaging with scalable 2D–3D deep learning framework for colorectal cancer detection

**DOI:** 10.1038/s41598-024-64917-5

**Published:** 2024-06-26

**Authors:** Willaim Oswald, Craig Browning, Ruthba Yasmin, Joshua Deal, Thomas C. Rich, Silas J. Leavesley, Na Gong

**Affiliations:** 1 Department of Electrical and Computer Engineering, University of South Alabama, Mobile Alabama, 36688 USA; 2 Department of Systems Engineering, University of South Alabama, Mobile, AL 36688 USA; 3 Department of Chemical and Biomolecular Engineering, University of South Alabama, Mobile, AL 36688 USA; 4 Nikon Instruments, Melville, NY 11747 USA; 5 Department of Pharmacology, University of South Alabama, Mobile, AL 36688 USA; 6 Center for Lung Biology, University of South Alabama, Mobile, AL 36688 USA

**Keywords:** Cancer imaging, Biomedical engineering

## Abstract

Colorectal cancer is one of the top contributors to cancer-related deaths in the United States, with over 100,000 estimated cases in 2020 and over 50,000 deaths. The most common screening technique is minimally invasive colonoscopy using either reflected white light endoscopy or narrow-band imaging. However, current imaging modalities have only moderate sensitivity and specificity for lesion detection. We have developed a novel fluorescence excitation-scanning hyperspectral imaging (HSI) approach to sample image and spectroscopic data simultaneously on microscope and endoscope platforms for enhanced diagnostic potential. Unfortunately, fluorescence excitation-scanning HSI datasets pose major challenges for data processing, interpretability, and classification due to their high dimensionality. Here, we present an end-to-end scalable Artificial Intelligence (AI) framework built for classification of excitation-scanning HSI microscopy data that provides accurate image classification and interpretability of the AI decision-making process. The developed AI framework is able to perform real-time HSI classification with different speed/classification performance trade-offs by tailoring the dimensionality of the dataset, supporting different dimensions of deep learning models, and varying the architecture of deep learning models. We have also incorporated tools to visualize the exact location of the lesion detected by the AI decision-making process and to provide heatmap-based pixel-by-pixel interpretability. In addition, our deep learning framework provides wavelength-dependent impact as a heatmap, which allows visualization of the contributions of HSI wavelength bands during the AI decision-making process. This framework is well-suited for HSI microscope and endoscope platforms, where real-time analysis and visualization of classification results are required by clinicians.

## Introduction

Colorectal cancer is one of the most common sources of cancer related deaths in the United States, with a lifetime risk of 4.1% (women)–4.4% (men)^1^. While at home screening tests are now available, endoscopic colonoscopy remains the gold standard for colorectal cancer screening, biopsy for pathologic diagnosis, and an important step in staging. The standard modality for endoscopic screening is white light endoscopy. Several alternative imaging modalities have also been developed to aid in visualization, including narrow band imaging, autofluorescence imaging, and chromoendoscopy. These alternative modalities can provide enhanced contrast of specific features, such as vasculature. Recent endoscopic technology developments, such as blue light endoscopy^2,3^, and computer-aided artificial intelligence (AI)-based detection^4,5^, have also been developed to provide improved visualization and detection capabilities. However, there remains a need for further development of endoscopic imaging modalities that provide enhanced visualization, especially of flat or depressed lesions, and improved detection accuracy. Hyperspectral imaging (HSI) is a spectroscopic imaging technique that provides 2-dimensional spatial image over a range of wavelengths (3rd dimension), where the additional spectroscopic data can be used for chemical and molecular analysis of cells and tissues. HSI holds great promise for increasing the capabilities and accuracy of clinical diagnostic instruments and prototype platforms have been developed for dermatology^6^, ophthalmology^7^, surgical scenarios^8,9^, and endoscopes^10–13^.

Unfortunately, typical HSI technologies achieve spectroscopic sampling by filtering reflected light or fluorescence emission, resulting in large losses of signal and corresponding long acquisition times. Hence, new HSI technologies are needed to enable real-time microscope and endoscope procedures. We have previously developed an alternative HSI technology, fluorescence excitation-scanning hyperspectral imaging, that provides improved signal strength and imaging speed and is well-suited for high-speed and real-time imaging applications^12,14,15^. We have previously demonstrated this approach for screening of excised colorectal cancer specimens on a microscope platform^12,16^.However, analysis of high-dimensional HSI datasets using traditional spectral unmixing or classification algorithms is difficult^17^, as clinical image data present greatly increased variability, compared to traditional HSI applications, such as remote sensing. Hence, new analysis approaches are needed to fully realize the diagnostic potential of excitation-scanning HSI, especially when implemented in real-time screening applications.

In recent years, deep learning-based image analysis approaches have been developed for traditional HSI applications. Due to their capability to automatically extract nonlinear features through a series of hierarchical layers, deep learning-based HSI analysis methods have obtained better classification performance as compared to traditional approaches, such as support vector machines (SVM)^18^, k-nearest neighbor algorithm (k-NN)^19^, and logistic regression^20^. For example, Halicek et al.^20^ presented a convolutional neural network (CNN), which consists of six convolutional layers and three fully connected layers to classify normal or cancerous head and neck tissue samples. The evaluation results of 50 patients showed that the developed CNN achieved an accuracy, sensitivity, and specificity of 80%, 81% and 78% respectively. Also, Li et al.^21^ developed a deep CNN to extract features from blood cell hyperspectral images and classify red and white cells, enabling an average accuracy of 93%. Very recently, high-dimensional deep learning such as three-dimensional (3D) deep learning approaches have been developed to fully utilize spectral-spatial properties of HSI, thereby achieving improved classification performance. As an example, Bengs et al.^22^ presented a CNN with 3D spatial-spectral convolutions called Densenet3D for laryngeal cancer detection that provided an average accuracy of 81%, sensitivity of 92% and specificity of 65%. In another recent study, Cihan et al.^23^ developed a 3D-CNN (CihanNet) with three convolutional layers to classify unhealthy and healthy neonates. Based on a dataset of 5,760 hypercubes, CihanNet achieved 98% accuracy, 97.22% sensitivity, and 98.78% specificity.

Although the above-mentioned deep learning-based HSI analysis approaches have achieved good classification performance, they suffer from two main limitations. First, existing work has sought to develop a specific deep learning architecture, which is usually designed for a specific task. The focus on optimizing the classification performance may limit the applicability in a variety of scenarios with different available computing resources or performance requirements, particularly considering various automatic classification tools in the clinical applications. Second, existing techniques have not focused on interpreting and visualizing the internal dynamics and decision-making process. The “black-box” nature of AI in general makes DNNs extremely difficult to interpret for medical professionals, which hinders the accessibility for future clinical applications. As such, saliency checks on the model are vital.

To overcome the two above-mentioned limitations of state-of-the art deep learning-based classification, in this study, we develop an end-to-end scalable HSI deep learning framework with real-time classification performance and visualization for analysis of fluorescence excitation-scanning HSI classification with different speed/classification performance trade-offs by tailoring the dimensionality of the dataset, supporting both 2D and 3D deep learning models, and varying the architecture of deep learning models. Also, it can visualize the exact location of the lesion if detected during the AI decision-making process. The framework can generate Gradient-weighted Class Activation Mapping (Grad-CAM) heatmaps, providing region-based interpretability. In addition, our deep learning framework can extract wavelength significance information from classification heatmaps, to visualize the contribution of different wavelengths of light, as seen from the DNN decision-making process. To the best of our knowledge, this study is the first to develop a DNN-based analysis framework for excitation-scanning HSI; it is also the first HSI DNN framework with speed/classification performance scalability and visualization for biomedical imaging analysis. The developed framework for classification of excitation-scanning HSI data has great potential to assist surgeons in the assessment of tumors and tumor margins, by providing real-time feedback during colonoscopy and endoscopic assisted procedures.

## Methods

### Tissue specimens

This study was approved by the University of South Alabama Institutional Review Board (IRB). All methods were performed in accordance with relevant guidelines and regulations. Human colorectal tissue specimens were obtained from the University of South Alabama, College of Medicine, Departments of Surgery and Pathology under an Institutional Review Board (IRB) approved protocol (IRB # 445452). All tissue specimens were obtained as deidentified residual specimens from standard-of-care surgical procedures, and only from procedures where sufficient tissue mass was resected so as to allow for procurement of specimens with no interruption to standard-of-care diagnosis. Hence, tissue specimens were obtained from deidentified residual tissues that were marked for disposal and informed consent was not required per IRB guidelines. A full description of tissue processing procedures can be found in^16^. In summary, fresh specimens were retrieved from the operating room and transferred to surgical pathology for assessment.

If deemed adequate for this study, a specimen of the primary tumor (neoplasm) was obtained along with a specimen of noninvolved (“normal”) tissue outside of the margin of the neoplasm. Specimens were placed in separate containers in phosphate buffered saline (PBS) and stored at 4 °C for ≤ 8 h. Prior to imaging, specimens were rinsed with PBS and cut into ~ 1-cm cubes. Specimens were mounted on a 25-mm-round coverslip in an AttoFluor holder (Life Technologies) and spectral image data were acquired using a custom excitation-scanning hyperspectral imaging microscope, using settings described below. For each specimen, a minimum of 3 fields of view (FOV) were acquired.

### Hyperspectral imaging

Imaging was conducted using a custom excitation-scanning hyperspectral imaging microscope platform^14,16^. The system consisted of an inverted widefield microscope base (Eclipse TE 2000-U, Nikon), a 20× objective (Plan Apo λ 20×/0.75 ∞/0.17 MRD00205, Nikon Instruments), and a back-illuminated EMCCD camera (Rolera em-c^2^, Q-Imaging) for image acquisition. A subset of images was acquired using a faster readout back-illuminated sCMOS camera (Prime 95B, Teledyne Photometrics). Spectral illumination was achieved using a broadband Xenon arc lamp (Titan 300, Sunoptics) and a tiltable filter wheel (VF-5, Sutter Instruments) containing an array of thin-film tunable filters (VersaChrome, Semrock Inc.). Spectrally selected excitation light was transmitted to the microscope through a liquid light guide. A long-pass dichroic beamsplitter (FF555-Di03-25 × 36) was used to reflect excitation light through the microscope objective to the sample and to transmit emitted fluorescence. A corresponding long-pass emission filter (BLP02 561R-25, Semrock Inc.) was used to detect fluorescence emission. Excitation-scanning hyperspectral image data were acquired from 360 to 550 nm in 5 nm increments (38 wavelength bands) by tuning the VF-5 filter wheel to each successive excitation wavelength band and acquiring an image of the resulting fluorescence emission. EMCCD camera acquisition settings were specified as 14-bit and 2 × 2 binning, resulting in an image size of 501 × 502 pixels.

**Figure 1:**
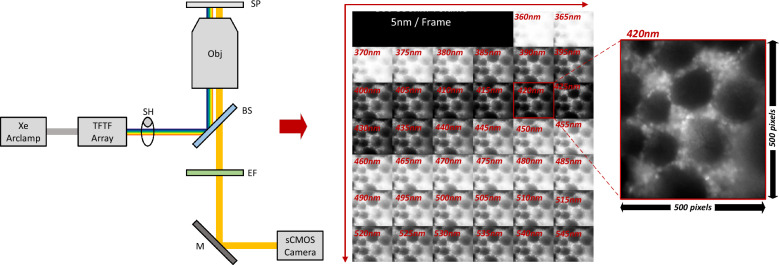
A single tissue sample captured using the MMOS Camera and settings as described. Frequencies are presented as panels, starting in the top left panel at 360nm, bottom right is 550nm. Each wavelength has an image resolution of 500 × 500.

sCMOS camera acquisition settings were specified as 16-bit acquisition and 2 × 2 binning, resulting in an image size of 600 × 600 pixels (Fig. 1). Pairs of lesional and non-lesional colorectal tissue specimens were imaged to compile the hyperspectral image dataset. Images were acquired from multiple FOV of each tissue specimen, so as to sample the diversity of spatial features within the specimen. Images were corrected to a flat spectral response using a fiber coupled spectrometer (QE65000, Ocean Optics), integrating sphere (FOIS-1, Ocean Optics), and NIST-traceable calibration lamp (LS-1-CAL, Ocean Optics) as described previously^14–16^. For the dataset, a total of 104 lesional and 112 non-lesional FOV were sampled^15^.

### Proposed 2D–3D deep learning framework

#### Top-down deep learning framework

**Figure 2:**
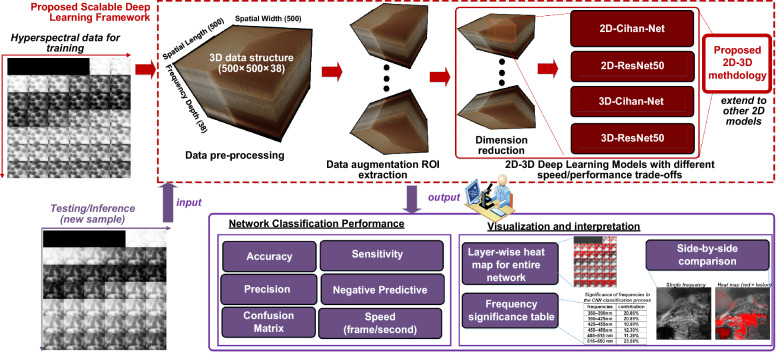
Proposed deep learning framework.

Figure 2 shows the developed 2D–3D deep learning framework. The collected hyperspectral imagery data, containing 38 frequencies (wavelength bands) was pre-processed with linear compression, histogram equalization and normalization and then combined into a 3D hyperspectral image volume with a size of 500 × 500 × 38. Using data augmentation and region-of-interest (ROI) extraction, a larger dataset with smaller spatial resolution images was obtained to train the neural network models. We used two different deep learning models (CihanNet^23^ and ResNet50^25^) which have shown to be effective for image classification. Another important reason these two architectures were chosen was they have very different computational complexities, which has potential to enable speed/classification trade-off opportunities. To further enhance scalability with different speed/classification performance trade-offs, we developed our deep learning framework using the following combined techniques: (i) varying the dimension of the dataset: the dataset was first processed with Principal Component Analysis (PCA) to produce different wavelength dimensions (e.g., 3, 8, 16, 32, 38 bands); (ii) varying the dimension of deep learning models: our framework supports both 2D and 3D architectures of models (CihanNet and ResNet50); and (iii) varying deep learning models: we propose a 2D-3D methodology to easily convert traditional 2D models to a 3D version, thereby extending our framework to general deep learning models. The details of each step will be discussed in the following sections.

#### Proposed 2D to 3D DNN methodology

In order to achieve scalable tradeoff levels between computational speed and classification accuracy, our deep learning framework supports both 2D and 3D deep learning architectures for any DNN model. Typically, traditional 2D architecture can enable higher speed classification due to the lower computation complexity, while 3D architecture can achieve higher accuracy due to more extracted features. To support 2D to 3D conversion of different models, we propose a lightweight but effective 2D-3D methodology, which can be used to extend our developed framework for general models.

For a specific DNN model, its 2D and 3D architectures have many similar features from a logical and computational perspective. This is because, only the convolutional layers inside the DNN require dimensionality information^24^. The 2D and 3D input data dimensions can be expressed as followed:

1 $$DataDimension2D=W\times H\times {C}_{1}$$

2 $$\text{DataDimension}3\text{D}=W\times H\times {B \times C}_{1}$$

where for both equations *W* and *H* are the spatial band information (width and height), *B* is the wavelength band, and ${C}_{1}$ the channel information generated by the filters of the convolutional layer. As the equations suggest, the 3D convolutional filters extract feature interactions between immediately adjacent channels, while 2D convolutional filters do not compute interactions between multiple wavelength bands within the convolutional filters, instead calculating spatial features within each wavelength band. This results in much faster computation for 2D convolution, but important wavelength (frequency) interaction information may be lost. With different input data dimensions, 2D or 3D convolution is computed as^24^.

3 $$2D: {map}_{i,j}^{x,y}=f\left({\sum }_{m}{\sum }_{h=0}^{{H}_{i}-1}{\sum }_{w=0}^{{W}_{i}-1}{k}_{i,j,m}^{h,w} {map}_{\left(i-1\right),m}^{\left(x+h\right),(y+w)}+{b}_{i,j}\right)$$

4 $$3D: {V}_{i,j}^{x,y,z}=f\left({\sum }_{m}{\sum }_{h=0}^{{H}_{i}-1}{\sum }_{w=0}^{{W}_{i}-1}{\sum }_{c=0}^{{C}_{i}-1}{k}_{i,j,m}^{h,w,c} {V}_{\left(i-1\right),m}^{\left(x+h\right),\left(y+w\right),(z+c)}+{b}_{i,j}\right)$$

In Eqs. (3) and (4), ${k}_{i,j,m}^{h,w,(c)}$ represents a specific value (such as pixel value), at a specific spatial height and width position (*h*, *w*), and specific channel (c). The kernel map is denoted as ${map}_{i,j}^{x,y}$ and ${V}_{i,j}^{x,y,z}$ with 2D $\text{map}$ and 3D $\text{map}$ calculating the kernel map for 2D and 3D respectively; at a specific spatial height and width position (h, w), and specific channel (c). The kernel map is denoted as ${map}_{i,j}^{x,y,z}$; at a specific position (x,y,z), and the *n*th feature map of the previous layer (or input). ${b}_{i,j}$ and *f()* represents the bias and the activation function, respectively. Equations (3) and (4) also reveal that 2D convolution is substantially easier to compute, however it cannot extract feature information that exist between adjacent wavelength bands.

**Figure 3:**
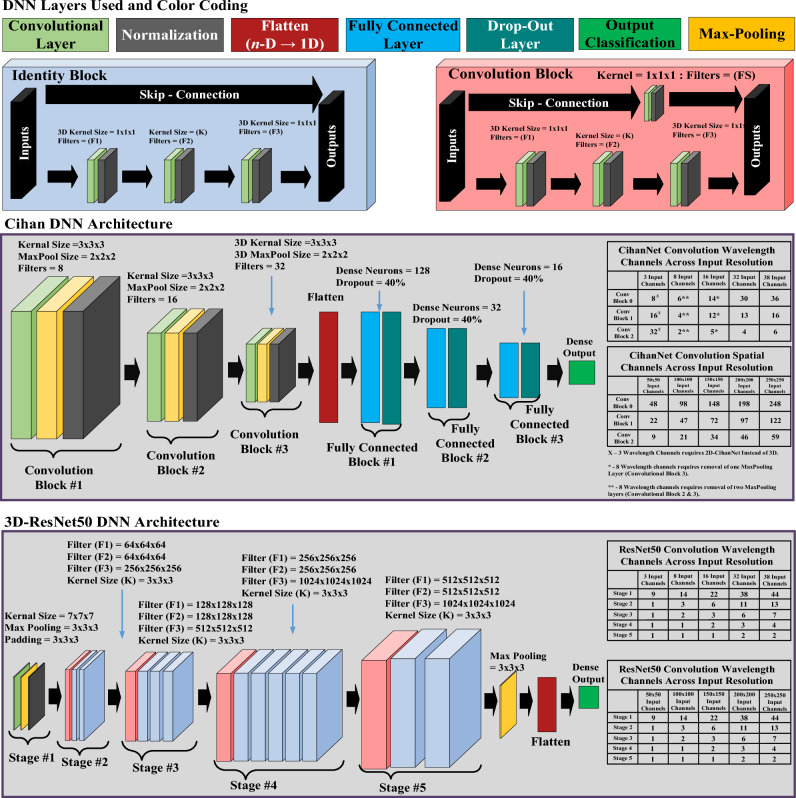
Deep learning network architectures. Sizing is consistent regardless of input resolution; the only exception is when wavelength channel from HSI volumes are too low (e.g., wavelengths channels =3), CihanNet requires 2D convolutional layers. Medium sized HSI images (e.g., wavelength channels =8 or 16) can still utilize 3D convolution, by removing MaxPooling layers.

To support the conversion of different traditional 2D models to 3D, we propose a simple methodology to adapt DNN architectures to accept a new number of input dimensions. To convert a 3D-DNN architecture into a 2D version, the 3D convolutional layers exchanged for 2D versions, and kernel sizes are adjusted to reflect the missing dimension. To convert a 2D-DNN architecture into a 3D convolutional layer, and the *K* × *K* kernel size of the layer is expanded to *K* × *K* × *K*. Here *K* is the kernel size of the specific convolutional layer. However, there does exist one exception to the rule for both 2D and 3D networks. Occasionally convolutional layers are immediately followed by max-pooling layers (Fig. 3). These max-pooling layers are intended to reduce computational complexity by pooling information together. It is possible that these pooling layers attempt to reduce the number of information channels into a negative value. This occurred in some of our initial tests when the number of wavelength bands or spatial dimensions was reduced. The approach to resolve this is to simply exclude the Max-Pooling layers, one at a time until some number of positive information channels exist throughout the entire network. As an example, if 3D-CihanNet is chosen as the DNN architecture, with 8 HSI frequencies, the Max-pooling layers in Convolutional Blocks #1 and #2 are omitted to avoid negative channels of information. Drop-out layers are present in DNN architectures to prevent overfitting^25^. ResNet does not include drop-out as overfitting was not observed in the original application.

####  Data pre-processing, augmentation and ROI extraction

Data preprocessing and cleaning were performed in several steps. First, all 3D volumes were scaled such that the image size was consistent. Scaling was applied using linear compression, reshaping each 3D volume to a size of 500 × 500 × 38. After scaling, pixel intensity histogram equalization was performed, followed by histogram normalization into 32-bit floating point values ranging between 0 and 1. The histogram equalization reduces the effects of variable intensity levels between samples or fields of view that arise due to varying tissue thickness and optical absorption and scattering.

After cleaning, data augmentation was applied to expand the dataset for neural network image classification. Different augmentation approaches such as rotating, mirroring, and mirror + rotate were performed along the spatial dimension. The 3D volumes were only rotated along spatial dimensions. Three rotations were performed, and along with the original orientation created the expanded dataset. The exact degrees of rotation were randomly selected for each sample, independent of other samples. The degree of rotation was defined as:

5 $${\theta }_{k}=k\left(90^\circ \right)\pm 45^\circ$$

where *k* represents an integer that is the loop number in the array (1, 2, 3). This resulted in three rotations of 90° ± 45°, 180° ± 45°, and 270° ± 45° as values of ${\uptheta }_{\text{k}}$. The 3D volume is rotated along a 2D axis along the spatial dimensions by this amount, with linear interpolation stretching applied to return the data to a size of 500 × 500 × 38. Wavelength bands are not altered in this way.

Finally, ROI extraction was performed along the spatial dimensions, maintaining all wavelength band information. The size of the ROI varied depending on the scalability setting desired. Classification accuracy and computation speed tradeoffs were considered as a function of ROI size. In our approach, ROIs of 50 × 50, 100 × 100, 150 × 150, 200 × 200, and 250 × 250 pixels were all investigated. The number of ROIs extracted was adjusted depending on the ROI size to avoid redundant overlapping of ROIs. Accordingly, the number of extracted ROIs *(*$${ROI}_{num})$$ was expressed using Eq. (6):

6 $${ROI}_{num}=\lfloor{\frac{{Img}_{size}}{{ROI}_{size}}}^{2}\rfloor-1$$

where ${Img}_{size}$ is the normalized volume size along a single spatial dimension, which is standardized to be 500 × 500 from the data cleaning process. ${ROI}_{size}$ is the variable ROI size for a specific DNN scale. Finally, ${ROI}_{num}$ of ROIs are extracted from each image with size (${ROI}_{size}$ × ${ROI}_{size}$ × ${f}_{PCA}$), from a random position within the full 3D volume. This process expands the dataset further, while also alleviating the probability of oversampling specific images. The process also has the benefit of not being a deterministic segmentation patterning technique that the DNN may learn to exploit. After data augmentation, samples were split into training and validation datasets, more details about data splitting for model training will be discussed in "Study design".

#### Dimension reduction

**Figure 4:**
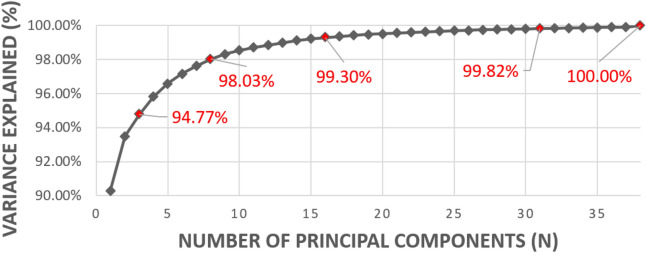
PCA Variance Ratio to justify number of principal components (PC) at each setting used in the proposed framework. Note at 38 frequencies, PCA is not performed.

To further increase tradeoff scalability between computational speed and classification accuracy for scalability, the proposed framework supports dimension reduction of the dataset. Specifically, Principal Component Analysis (PCA) was performed on the 3D volume along the wavelength dimension. PCA effectiveness was determined by the explained variance after the dimensionality was reduced. Figure 4 shows the dependence of information contained within a volumetric dataset on wavelength dimensionality—with three principal components (PC), 94.8% of the total variance was explained. As more PCs were considered, the explained variance of 95% or greater is deemed successful. In this study, we evaluated CNN performance both with and without PCA, utilizing 3, 8, 16, or 32 PCs with PCA, or the full 38 wavelength bands without PCA. The specific NN architecture that was used to classify images was adjusted to match the input image dimensionality. In other words, the first layer of the NN was scaled depending on both the image size from the ROI segmentation and the number of PCs. During this process, PCA was implemented using a singular value decomposition approach across the training augmented dataset.

#### Architecture of deep neural network models

As discussed earlier, two DNN models—ResNet50^26^ and CihanNet^23^ were included in the proposed framework. Specifically, the framework included an existing 3D-CihanNet^23^, existing 2D-ResNet50^25^, a newly converted 2D-CihanNet, as well as a new 3D-ResNet, using our developed 2D-3D methodology. The architecture details of 3D CihanNet and 3D-ResNet50 are shown in Fig. 3. As discussed earlier, these two architectures have both seen success in standard image classification tasks. ResNet50 represents a near fully convolutional NN, with only the final layer using fully connected softmax neurons for output signals. Alternatively, CihanNet begins with convolutional blocks, then flattens the DNN architecture for fully-connected blocks before the finals dense layer generates output.

It should be also noted that NN architectures involving recurrent layers were considered, however deemed unnecessary dude to image-based datasets, unlike timing sensitive data such as videos. The absence of a time dimension in this dataset makes a Recurrent Neural Network (RNN) overly complex for negligible gains in accuracy. For the reason, other HSI based DNN architectures such as^27,28^ were not included in our framework. However, using our developed 2D-3D methodology, different DNN models (including RNN), can be converted to 3D architecture and added to our proposed deep learning framework to support different applications.

###  Study design

**Figure 5:**
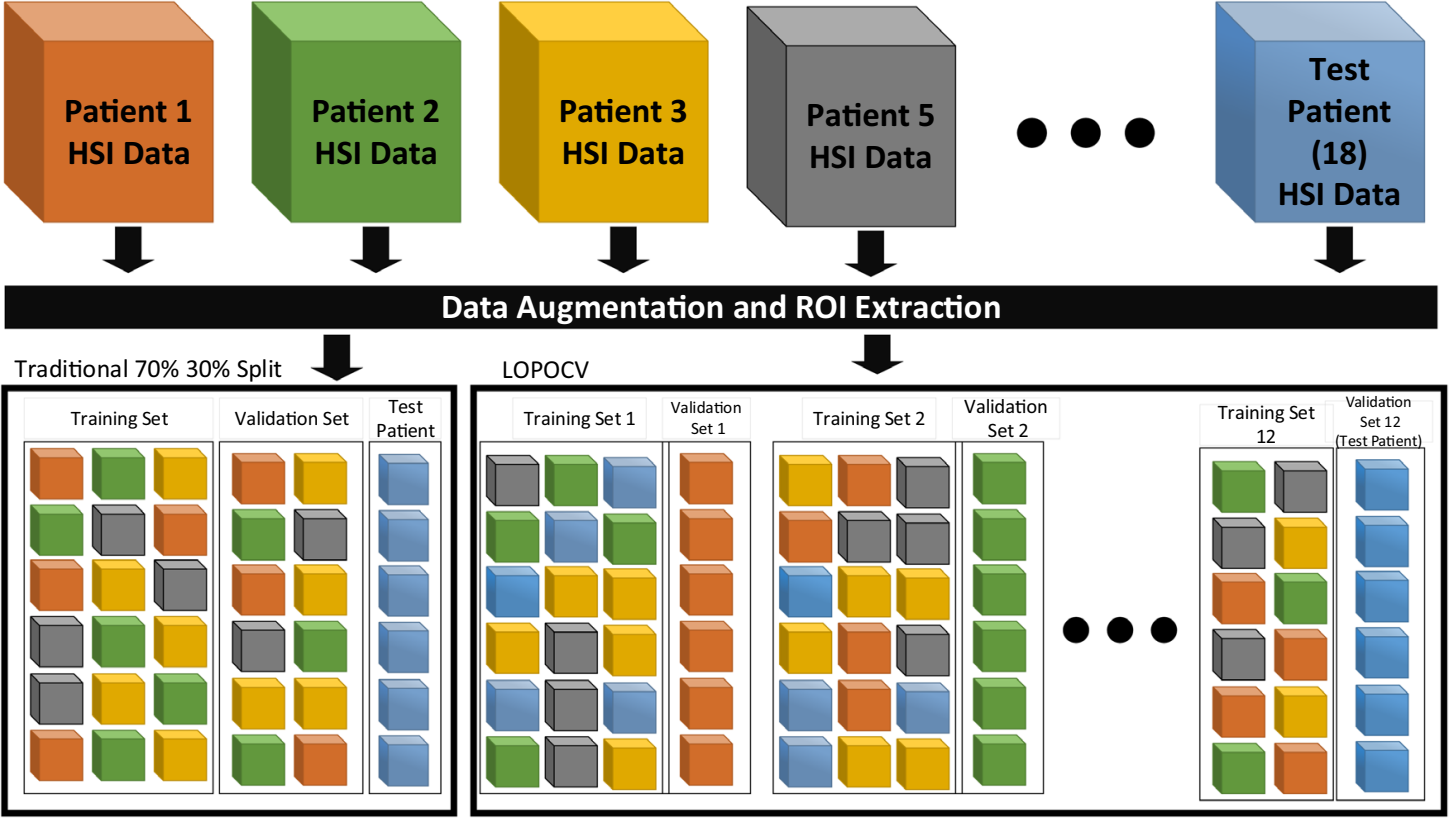
Two different data partitioning strategies.

Our study adopted two different data partitioning strategies, which are both widely used in state-of-the art, as illustrated in Fig. 5. We first used the traditional widely-adopted training/validation dataset splitting process as well as independent patient for external validation. Specifically, the image dataset consisted of images acquired from pairs of lesional and non-lesional tissue specimens obtained from 11 patients, containing 179 fields of view (FOV). The dataset was refined to 69 lesional FOVs and 70 healthy FOVs by ignoring FOVs that were acquired as part of the microscope calibration process. After cleaning and augmentation, the 179 samples were then split into training (70%) and validation datasets (30%)^29^. It should be emphasized that the dataset was split before PCA, as PCA itself requires training, and thus should not be exposed to the validation dataset. To validate that the framework operated correctly, and could accurately predict a sample never before seen, the NN was not permitted to learn from the validation dataset, only make predictions upon it. To further verify the generalization capacity of our deep learning framework and ensure the reliability and accuracy of the results, an additional pair of independent test specimens were obtained from the Cooperative Human Tissue Network (CHTN) that were not part of the original image dataset. The additional deidentified specimen pair was imaged using similar acquisition settings as the current image dataset.

Considering the practical clinical diagnosis, the AI framework will be used to classify new patients’ data. Therefore, in the second data partitioning strategy, the training dataset and test dataset do not share any same patients. To achieve it, we were able to obtain six additional clinical specimen pairs through partnership with the Cooperative Human Tissue Network (CHTN). We added the new six patients to the existing twelve patients, bringing the total number of patients to 18, and the total number of FOV collected to 216 (including 112 lesional and 104 healthy FOVs). We used the data from all 18 patients to employed leave-one-patient-out cross-validation (LOPOCV) strategy^30^. Specifically, for each LOPOCV run, the data from one patient was used as the test set and all other patients’ data was used as training set. LOPOCV is a variant of the *K*-fold cross-validation, in which *K* is set to be the number of the patients, i.e., 18 in our study.

Using both data partitioning strategies, multiple key metrics were used to evaluate classification performance, including Sensitivity, Specificity, Miss Rate, False Discovery, and Accuracy. Each metric was measured as follows:

7 $$Sensitivity=\frac{TP}{TP+FP}$$

8 $$Specificity=\frac{TN}{TN+FN}$$

9 $$Miss\, Rate=\frac{FN}{TP+FN}$$

10 $$False\, Discovery \,Rate=\frac{FP}{TP+FP}$$

11 $$Accuracy=\frac{TP+TN}{TP+FP+TN+FN}$$

where *TP* represents True-Positive images, lesional samples correctly classified; *TN* represents True-Negative images, healthy samples correctly classified; *FP* represents the False-Positive samples; and *FN* represents the False-Negative samples classified by the framework.

Each model was trained for 50 epochs, with an initial learning rate of 0.001, with an exponential decay of 0.8 applied every 10 epochs. In addition to classification performance, the computation speed was evaluated. This study was conducted on multiple compute infrastructures, ranging from a desktop workstation, a Compute Server, and high performance computing (HPC) clusters. In terms of computing speed evaluations, we present the compute speed within the server, and limited the hardware to only accessing a single GPU for acceleration. For this study, the system utilized an Intel Xeon Gold 5218 CPU, paired with an Nvidia A6000 GPU, and 380 GB of 3200 MHz DDR4 memory, running on Ubuntu 20.04 LTS, inside Python 3.9, Tensorflow 2.4.1. Speed may vary drastically depending on software version numbers, so the total number of parameters for each DNN architecture are also presented to estimate on different systems.

## Results

### Classification performance

#### Validation accuracy of DNN models

With standard 70%/30% training/validation dataset splitting, the classification accuracy of different DNN models was observed to scale with relationship to the selected architecture and input ROI size (Table 1). Two-dimensional CNN models could be used when the number of PCs was 3, whereas 3D models were required as the number of PCs increased. It can be seen that, although using 3 PCs accounts for 94.7% of the variance information contained within the original 38 bands (Fig. 4), the 2D-CihanNet and the 2D-ResNet50 yielded much lower classification performance as compared to their 3D versions. Specifically, 2D-CihanNet yields dissatisfactory classification performance with only 57.2–60.54% validation accuracy, while 2D-ResNet50 shows relatively better performance with the highest accuracy of 83.3%. These results indicate that the statistical variance accounted for during PCA compression is not a useful metric in determining if important information for classification is retained during the compression. In fact, PCA compression needs to apply no lower than 8PCs for CihanNet in order to achieve over 60.53% accuracy. It can be seen that across the various settings, ResNet50 achieves higher accuracies as compared to the CihanNet architectures, with accuracy as high as 99.43%. Also, across the various number of wavelength bands, there was a trend that ROI sizing of 100 × 100 produced the highest accuracy. A spatial resolution of this size indicates that the total number of samples used for training is important. A tradeoff between the total number of ROIs and ROI image resolution is implemented to prevent oversampling and is detailed in the Methods section. Using too few training samples results in difficulty for the NN to generalize a solution, leading to lower accuracies. This leads to a general trend of an image size of 100 × 100 pixels that is optimal to achieve the highest validation accuracy (Table 1).

**Table 1:** Training and validation accuracy for all DNN architectures presented, at all frequencies and resolutions.

ROI size	#Bands (after PCA)	DNN type^a^	#Samples (Train + Val.)	CihanNet (2D and 3D)^a^		ResNet50 (2D and 3D)^a^	
				Train accuracy^b,c^ (%)	Validation accuracy^b,c^ (%)	Train accuracy (%)	Validation accuracy (%)
*50* × *50*	3	2D	106,326	56.03	57.20	69.94	58.03
*100* × *100*	3	2D	25,776	55.37	50.00	94.22	83.31
*150* × *150*	3	2D	10,740	57.46	55.55	93.72	73.56
*200* × *200*	3	2D	5370	59.97	60.38	91.45	68.97
*250* × *250*	3	2D	3222	59.29	60.54	80.62	62.38
50 × 50	8	3D	106,326	96.62	93.68	99.27	99.37
**100 × 100**	**8**	**3D**	**25,776**	**96.51**	**94.57**	99.71	95.68
150 × 150	8	3D	10,740	80.57	85.87	99.02	99.46
200 × 200	8	3D	5370	50.38	53.99	97.94	98.40
250 × 250	8	3D	3222	50.65	50.48	97.19	96.17
50 × 50	16	3D	106,326	98.86	99.12	99.45	99.43
100 × 100	16	3D	25,776	99.24	84.28	99.81	98.30
150 × 150	16	3D	10,740	78.59	79.70	99.49	99.52
200 × 200	16	3D	5370	84.37	84.03	99.96	99.04
250 × 250	16	3D	3222	84.92	86.90	99.79	99.96
50 × 50	32	3D	106,326	98.97	99.12	99.61	99.17
100 × 100	32	3D	25,776	99.67	99.29	99.95	98.96
150 × 150	32	3D	10,740	99.43	99.04	99.90	99.16
200 × 200	32	3D	5370	98.42	97.44	99.73	99.04
250 × 250	32	3D	3222	99.38	98.56	99.86	99.68
50 × 50	38	3D	106,326	99.19	99.25	99.63	99.22
**100 × 100**	**38**	**3D**	**25,776**	99.73	91.43	**99.87**	**99.43**
150 × 150	38	3D	10,740	98.43	97.49	99.74	99.04
200 × 200	38	3D	5370	90.61	89.14	99.31	99.84
250 × 250	38	3D	3222	93.76	92.65	99.52	98.40

*Tests used the original 179 FOVs, with the training and validation datasets split (70/30%).

^a^Results for 2D models shaded with number of frequencies (after PCA) equals 3.

^b^ChianNet with16 and 32 bands required the removal of max pooling layer #1 to avoid negative dimensions.

^c^ChianNet with 8 bands required the removal of max pooling layers #1 & #2 to avoid negative dimensions.

Specific settings discussed in future sections are highlighted in bold.

It should also be noted that there is a general trend in Table 1, for training data to achieve the highest accuracy in both architectures as the number of wavelength bands preserved after PCA increases. This indicates a general trend that high accuracies are to be expected as either a 32 or 28 wavelength band volume. Another observation is that training accuracy correlates well with validation accuracy. This implies that the DNNN was able to generalize a solution, suggesting the system will perform reliably for similar samples never seen before. Only top performing NNs from each epoch in training were captured. For this reason, there does still exist variation in DNN accuracy, due to the randomness involved with training a NN, which is the reason why accuracy varies slightly as compared to similar settings (Table 1 and Table 3). For stable high accuracy NN architectures, the optimal performance was achieved with 3D-ResNet50 with an input resolution of 100 × 100x38 and excluding PCA reduction.

####  Computation speed

Computation speed is dependent on multiple factors such as model architecture, model size, input size, and available computing resources. Based on our computing platform detailed in "Study design ", it can be seen that CihanNet has considerably fewer trainable weights in the architecture, compared to ResNet50, resulting in increased speed, as reflected by frames per second (FPS) values. Hence, there is a trade-off between computational complexity and the speed at which the NN can process images. On average CihanNet performs 63FPS faster than the ResNet50 counterpart given the same input resolution. These measurements were obtained on our hardware and may vary. Depending on use case, the speedup may allow more common hardware to be utilized instead of specialized hardware. As Not all applications will have access to servers in the cloud. For these reasons CihanNet is useful, as the accuracy drop is minimal with some settings. Specifically, CihanNet with a resolution of 100 × 100 × 8 performs with 94.57% accuracy, using only ~ 1/5th of the total number of trainable weights as compared to the High-Accuracy 3D-ResNet50 model (Table 1). Based on our computing platform, this down-sizing of the DNN yielded a 104 FPS increase in computation speed, making this a DNN that is well-suited for minimal hardware and edge devices.

The speed of the entire classification system is important to consider for applications outside of classification of this image dataset, for example analysis of large databases for mass screening. In these cases, computation could require several weeks to months, and effective approaches to speed up the analysis could produce large improvements in terms of computational time and cost. Alternatively, it is likely that in the near future, DNN analysis will need to be performed rapidly during endoscopic screening, in which case it would be necessary to provide classification results in real time for clinical use. Classification results for ROIs could provide clinicians with valuable flags or ques during colonoscopy or endoscopic surgery. Computation speed varies depending on the scalable ROI size and PCA components needed. This variance in speed can be calculated by adding the computation times of PCA and DNN classification (Table 2). The total computation time shown indicates the FPS value of classification the system can perform, given the computing platform.

**Table 2:** Speed comparison for different DNN architectures.

ROI size	# Bands (after PCA)	PCA comp. time (ms)	DNN type^a^	CihanNet (2D and 3D)^2^			ResNet50 (2D and 3D)		
				# of trainable weights	DNN comp. time (ms)	FPS	# of trainable weights	DNN comp. time (ms)	FPS
*50* × *50*	3	0.6	2D	–	–	–	76,625	11	86.2
*100* × *100*	3	2.3	2D	–	–	–	420,689	8	97.1
*150* × *150*	3	7.6	2D	–	–	–	1,194,833	8	64.1
*200* × *200*	3	16.2	2D	–	–	–	2,177,873	9	39.7
*250* × *250*	3	29.1	2D	–	–	–	3,455,825	10	25.6
50 × 50	8	0.7	3D	2,004,929	5	175.4	46,136,881	11	85.5
**100 × 100**	**8**	**2.7**	**3D**	**9,070,529**	**3**	**175.4**	46,147,121	11	73.0
150 × 150	8	6.7	3D	21,256,129	4	93.5	46,161,457	12	53.5
200 × 200	8	18.7	3D	38,561,729	5	42.2	46,179,889	15	29.7
250 × 250	8	28.6	3D	60,987,329	8	27.3	46,202,417	18	21.5
50 × 50	16	0.7	3D	841,665	5	175.4	46,137,011	11	85.5
100 × 100	16	3.3	3D	4,356,033	4	137.0	46,147,251	12	65.4
150 × 150	16	8.4	3D	10,057,665	6	69.4	46,161,587	15	42.7
200 × 200	16	23	3D	18,896,833	9	31.3	46,180,019	21	22.7
250 × 250	16	37.8	3D	29,513,665	14	19.3	46,202,547	28	15.2
50 × 50	32	1.1	3D	153,537	6	140.8	46,137,257	12	76.3
100 × 100	32	4.1	3D	841,665	5	109.9	46,147,497	14	55.2
150 × 150	32	11.3	3D	2,389,953	10	46.9	46,161,833	22	30.0
200 × 200	32	36.9	3D	4,356,033	17	18.6	46,180,265	31	14.7
250 × 250	32	46.5	3D	6,911,937	22	14.6	46,202,793	47	10.7
50 × 50	38	0	3D	219,185	3	333.3	46,137,365	12	83.3
**100 × 100**	**38**	**0**	**3D**	1,251,265	6	166.7	**46,147,605**	**14**	**71.4**
150 × 150	38	0	3D	3,573,697	12	83.3	46,161,941	26	38.5
200 × 200	38	0	3D	6,522,817	17	58.8	46,180,373	37	27.0
250 × 250	38	0	3D	10,356,673	29	34.5	46,202,901	56	17.9

^a^Results for 2D models shaded with number of frequencies (after PCA) equals 3.

^b^Speed of 2D-Cihan Net was not included due to their dissatisfactory classification performance.

Specific settings discussed in future sections are highlighted in bold.

####  Independent test

We further assessed the generalization capacity of our framework using an independent test dataset which was collected from samples taken from a new patient (Table 3). Results show that 3D-ResNet50 performs reliably with above 99% classification accuracy while the test accuracy approaches the validation accuracy, indicating the reliability of the developed framework. Another observation from Table 3 indicates that the DNN tends to produce either a high miss-rate or a high false discovery rate for misclassified images. These metrics may be useful when determining the solution strategy for other systems, if for example an overly sensitive system with a high false discovery rate is preferable. The results also show that the CihanNet architecture trains with unpredictable variance, approximately gaining or losing ~ 10% in classification performance between minor changes in training parameters. By contrast, ResNet50 varies ~ 2% in classification accuracy. This investigation showed a larger difference in accuracy separation between CihanNet and ResNet50. With CihanNet showing full failure when number of bands is 3, and unstable success with number of bands as 8.

**Table 3:** Independent test.

*# f*	CihanNet 100 × 100 × 18					ResNet50 100 × 100 × 38				
	Test accuracy (%)	Test sensitivity (%)	Test specificity (%)	Test miss rate (%)	Test false discovery rate (%)	Test accuracy (%)	Test sensitivity (%)	Test specificity (%)	Test miss rate (%)	Test false discovery rate (%)
3	–	–	–	–	–	98.13	96.31	99.94	0.03	3.69
8	**89.81%**	**92.69**	**86.94**	**7.27**	**7.31**	99.69	100.0	99.38	0.31	0.00
16	82.47	99.94	65.00	21.22	0.06	98.09	98.75	97.44	1.31	1.25
32	93.03	99.69	86.38	7.32	0.31	96.00	100.0	92.00	4.17	0.00
38	83.33	66.67	100.0	0.00	33.33	**100.0**	**100.0**	**100.0**	**0.00**	**0.00**

Specific settings discussed in future sections are presented in bold.

#### LOPOCV

LOPOCV was performed on data from all 18 patients (including the new six patients to the existing twelve patients), to verify classification on unseen patients in the clinical setting. Table 4 detailed patient-by-patient classification performance. Also, to present DNN performance accurately, the average performance with standard deviation of model performance from all LOPOCV are also presented. As shown, CihanNet with a resolution of 100 × 100 × 8 achieved 88.72 ± 3.34% training accuracy, and 83.04 ± 5.87% validation accuracy, while 3D-ResNet50 with an image resolution of 100 × 100 × 38 achieved 95.58 ± 1.78% training accuracy, and 93.01 ± 2.88% validation accuracy. It can be seen from Table 4 that these architectures can perform well given any newly introduced patient. As compared to the first data partition process, both models demonstrate a drop in performance of ~ 10% in the overall accuracy and this trend is consistent with the findings of previous studies using Raman spectroscopy data for colon cancer detection^31^.

**Table 4:** LOPOCV results across the top performing architectures.

LOPOCV Run #	CihanNet 100 × 100 × 8		ResNet50 100 × 100 × 38	
	Training accuracy (%)	Validation accuracy (%)	Training accuracy (%)	Validation accuracy (%)
1	89.62	92.03	94.78	94.41
2	91.49	87.59	93.82	88.90
3	90.61	78.05	93.24	93.30
4	85.28	81.34	95.90	91.74
5	84.41	79.60	98.34	90.07
6	93.05	93.22	97.72	95.84
7	88.21	82.82	97.54	95.91
8	84.69	79.00	95.83	91.20
9	87.47	88.40	95.15	97.37
10	84.60	83.86	94.83	96.24
11	91.72	86.09	99.30	95.83
12	82.30	81.76	94.68	96.11
13	89.76	80.65	93.39	91.74
14	95.15	73.32	94.51	87.38
15	90.20	79.98	92.66	94.99
16	87.21	79.42	97.01	90.10
17	91.00	73.93	95.46	92.87
18	90.20	93.68	96.36	90.25
Average	88.72	83.04	95.58	93.01
Standard deviation	3.34	5.87	1.78	2.88

**Figure 6:**
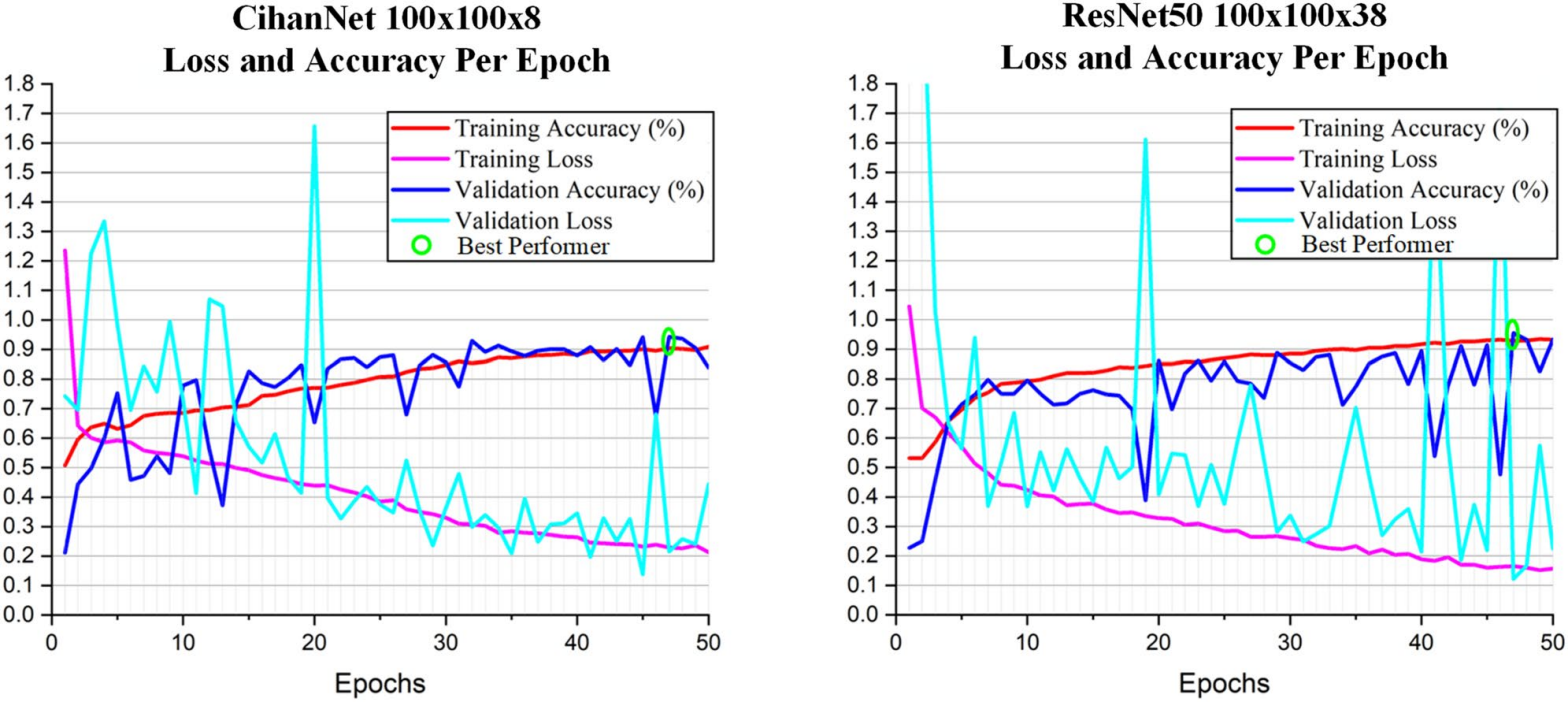
ResNet50 and CihanNet loss and accuracy across LOPOCV (Run #1). It shows spikes of validation accuracy loss at random points for training. The best Performer is selected by identifying the highest validation accuracy, when training accuracy stays within ± 1% of peak value. The trend shows a general increase in accuracy.

It can also be observed from Table 4 that across all of the LOPOCV runs, there are notable anomalies in which the validation accuracy drops, and validation loss rises. Additionally, validation accuracies do not always converge to training accuracies. Accordingly, specific runs (LOPOCV Run #1) for both ResNet50 and CihanNet are further studied and the results are shown in Fig. 6. From the figure it can be concluded that general trends of increasing accuracy for both the training and validation sets. The validation accuracy does present notable variability in classification performance on a per-epoch basis. This variability is notably higher for ResNet50 as compared to CihanNet. The source of the volatility is theorized to originate from the limited dataset of 216 FOVs, from the 18 patients. Specifically, in both architectures, there are some LOPOCV run outliers that achieved far lower accuracies than the reported average (e.g., Run #14). These patients performing poorly with both architectures reinforces the theory of the limited dataset being a major constraint, as this patients FOVs likely had features that are not well captured in the other patients’ tissue. With this extreme outlier however, the high accuracy model still achieved an 87.38% validation accuracy.

### Interpretability & visualization

#### Grad-CAM heatmaps

**Figure 7:**
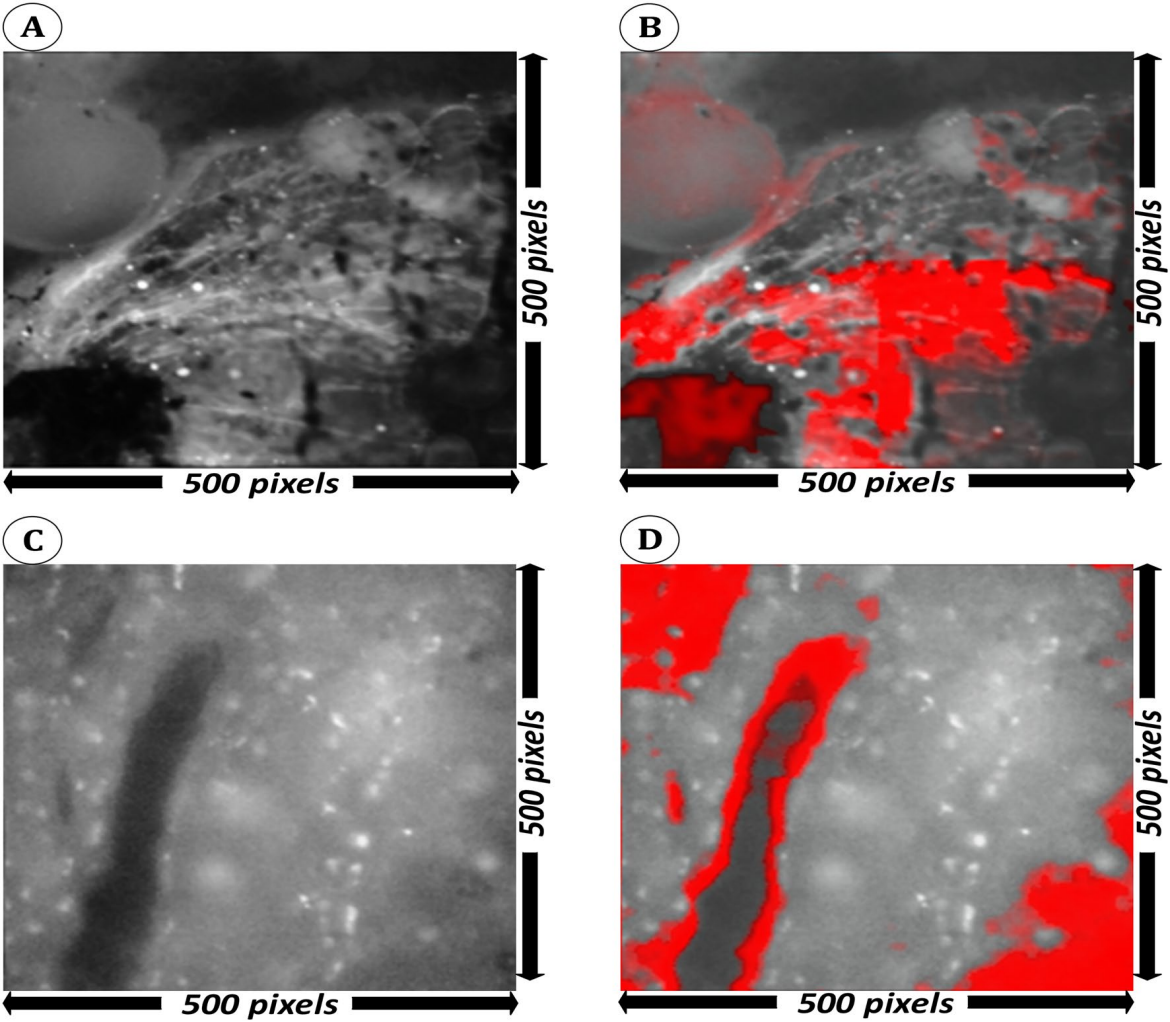
Heatmap visualization of a side-to-side comparison (single channel). Images (**A,C**) are the original images captured Images in gray scale. (**B,D**) Have the Grad-CAM heatmap overlaid and colored red. Each wavelength has an image resolution of 500 × 500.

**Figure 8:**
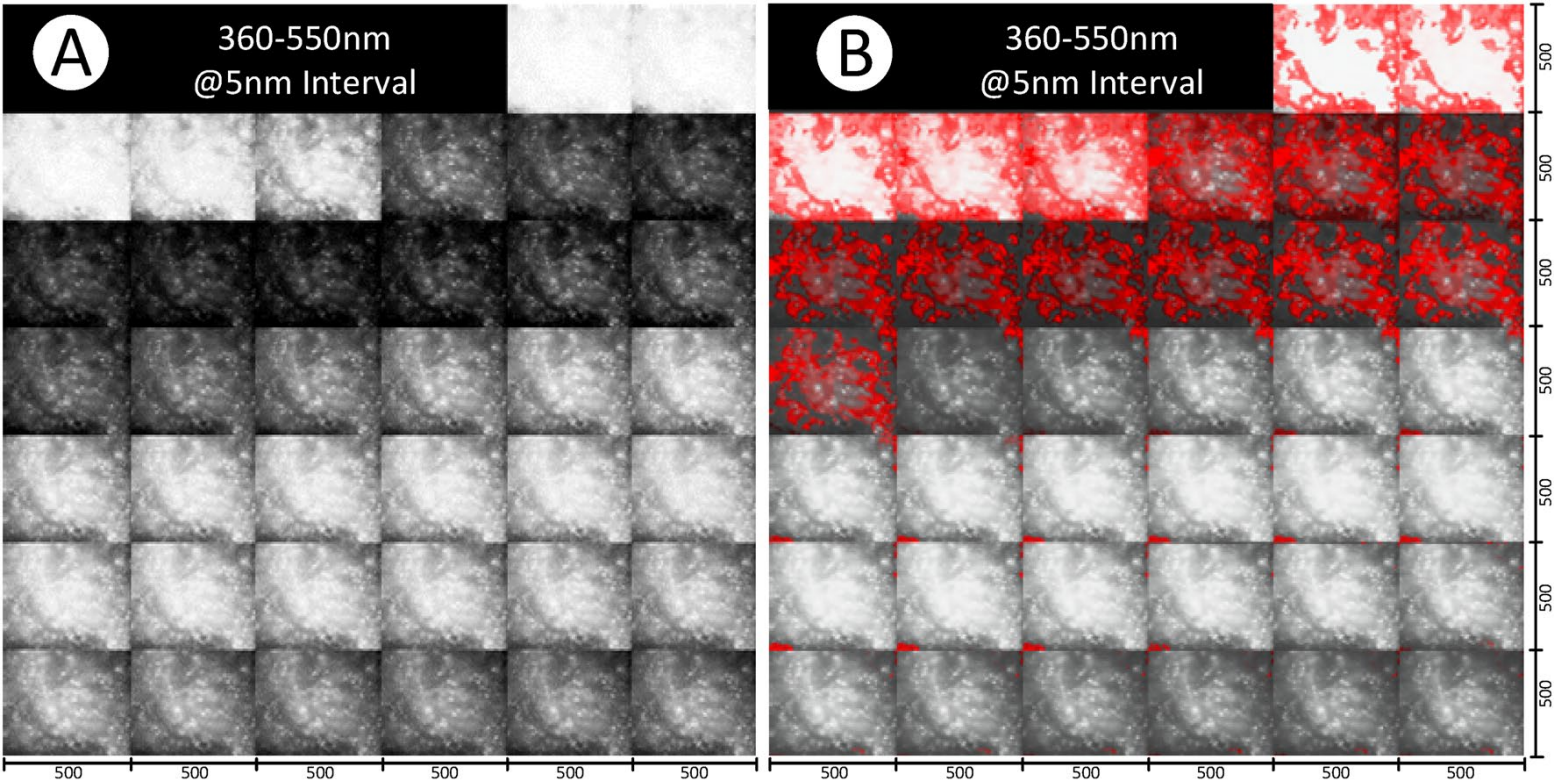
A side by side comparison of the original HSI 3D volume (**A**), and the Grad-CAM heatmap overlay (**B**). The heatmap is stitched together from four volumes of size 250 × 250 × 38 for the DNN to process. The resulting heatmap shows pixels deemed signs of a lesion, indicated by red intensity. Brighter reds indicate higher confidence in signs of lesion. Grad-CAM heatmap shown comes from the CihanNet Architecture. Images have a resolution of 500 × 500 × 38.

**Figure 9:**
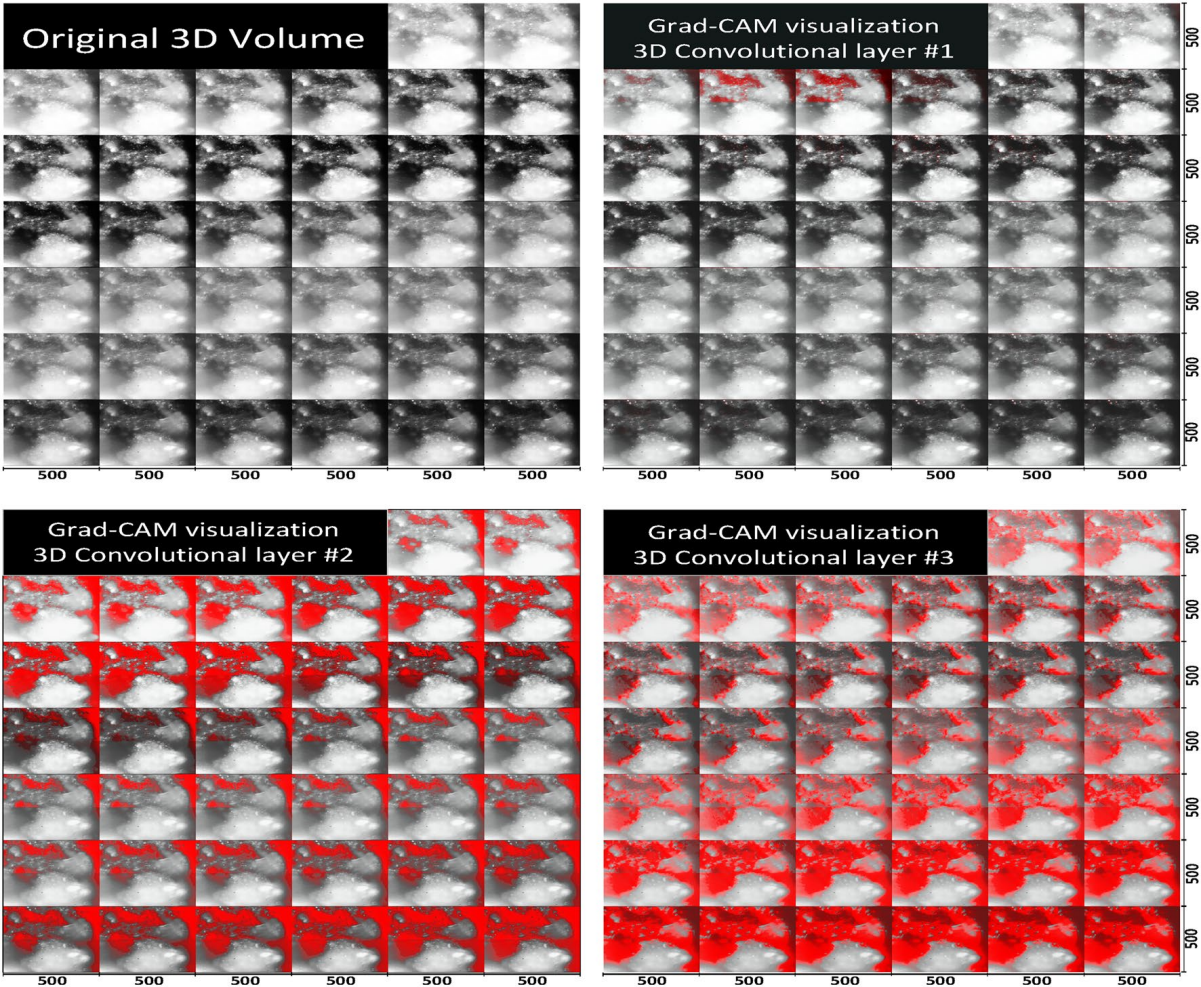
Heatmap visualization of lesional sample across the three 3D-CNN layers from CihanNet. Red areas indicated a stronger confidence that a lesion was present. Images have a resolution of 500 × 500 × 38.

In this work, we have used Grad-CAM as an initial approach to provide interpretability to some extent, which may be useful as an approximate visualization what wavelength bands and what spatial regions of an image may make the highest contribution towards the image being classified as cancerous. In the generated Grad-CAM heatmaps, red area indicates a stronger confidence that a lesion was present. The band-specific heatmaps of CihanNet are illustrated in Fig. 7 comparing two original images (A and C) and their heatmaps (B and D). The heatmap images show which pixels lead to a positive NN classification of colorectal cancer. A color lookup table was applied such that bright red pixels indicate a high confidence for a positive decision, while dull red colors indicate a low confidence. By taking all wavelengths into account, Fig. 8 compares the original HSI 3D volume (A) and the Grad-CAM heatmap overlay (B). The heatmap was stitched together from four volumes of size 250 × 250 × 38 for the DNN to process. Expanding on this, one full HSI image and its combined heatmap are shown (Fig. 9). It can be seen that the DNN is weighted to favor lower wavelength band information (B), leaving the majority of the high frequency information unneeded for classification. Further investigations into Grad-CAM yield (Fig. 8), which shows layer-wise heatmaps for the different layers in the network. The red areas in Layer #1 indicated lesional structures that are detectable with a single convolution, and thus are easily located. By Convolutional layer #2, most of the areas are well defined, with the highest confidence being that of layer #3. In general, it is common practice to inspect the last convolutional layer for heatmap interpretation, as this layer considers all information from previous layers. Band-dependent heatmap images were also combined into a single heatmap that facilitates visualization of any pixel contributing to a positive classification decision. It is worthy to emphasize that, Grad-CAM heatmaps provide huristic interpretability into ROIs within the original HSI volumes the DNN deem important for classificaiton purposes. Future work will include enabeling the DNNs to process in real-time as the data is being collected to investigate spesific features these heatmaps fixate upon.

#### Frequency significance table

Based on the overall Grad-CAM heatmap, the framework can further extract the contribution of different bands to the DNN decision-making process (Table 5). It can be observed that specific wavelength ranges contribute more information in detecting a lesion. Specifically, low wavelength (360–425 nm) represents 41.75% of the decision-making information. This analysis indicates that there are features specific to this wavelength band range that the DNN is discovering in the feature extraction process inside the convolutional layers. It should be emphasized that, our Grad-CAM based analysis is intended as a rough indication of bands or areas in an image that are of interest, and not a final visualization strategy that could be implemented in the clinic. Future work will investigate specific features explaining why the DNN focuses on low wavelengths, and investigate DNN performance as various HSI bands are omitted from the original HSI collection process.

**Table 5:** Grad-CAM Heatmap traceability back to original wavelength bands.

Frequencies (approximation) (nm)	Importance (%)
360–390	20.86
390–425	20.89
425–455	10.99
455–485	12.30
485–515	11.39
515–550	23.58

Wavelengths ranging 360–425 nm (low), 425–485 nm (middle), 575–610 nm (high) contribute 41.75%, 23.29%, and 34.97% of the information to the DNN decision-making process respectively. Showing a greater importance to low and high wavelengths.

## Discussion

In general, for a DNN system, any reduction in initial size of the first layer can carry significant impacts throughout the entire network size. Hence, small input images vastly increase processing speed and lower hardware requirements. However, this reduction comes with the additional cost of PCA for dimension reduction, which must be processed for each image. This is the reason why 38 band computation time is surprisingly faster than 32 bands, as 38 bands does not require PCA (Table 1). This framework presents two optimal solutions for high speed and high accuracy, depending on the desired system requirements, as shown in Table 4. For systems that need to operate at very high speeds, CihanNet with PCA = 8 is recommended. For high-accuracy applications, 3D-ResNet50 without PCA is verified to work with patient never before seen via LOPOCV with 95.58 ± 1.78% training accuracy, and 93.01 ± 2.88% validation accuracy, while CihanNet achieves 88.72 ± 3.34% training and 83.04 ± 5.87% validation accuracy with Gad-CAM heatmaps presenting a heuristic interpretation into the important features presented in the HSI system.

For speed sensitive systems, such as in real-time clinical procedures, it may be advantageous to use the CihanNet architecture, especially for speeds above 150FPS. The fast computation time of CihanNet may also lend itself to implementation on low-end electronic devices, where it may not be practical to use 3D-ResNet 50. However, a compromise of ~ 11% decreased test accuracy is associated. If the task is to rapidly process images on a resource-constrained computing platform (e.g., for real-time endoscopic screening), PCA compression to 3 PC bands can be used while still providing some accuracy and while greatly reducing computing demands. The traditional 2D-ResNet50 architecture with an image size of 150 × 150 is a good option, which can achieve a fast-processing speed (71.4 fps on our platform), with satisfactory classification performance.

If real-time results are not desired, lower-end computing platforms may be used while still achieving high accuracy, albeit at the expense of longer computation time. In this case, the 3D-ResNet50 architecture can provide a very high accuracy. An advantage of this approach is that a general-purpose low-cost CPU can be used to generate the Grad-CAM heatmaps, but DNN hardware accelerators e.g., Graphics Processing Units (GPU), Field Programmable Gate arrays (FPGA), or an application specific integrated circuit (ASIC), can be used to increase speed.

## Data Availability

Data available from the corresponding author (Dr. Silas J. Leavesley, leavesley@southalabama.edu) upon request.
